# Left Ventricular Global Function Index: A Potential Predictor of Mortality and Major Adverse Cardiovascular Events in NSTEMI Patients

**DOI:** 10.3390/medicina61030487

**Published:** 2025-03-11

**Authors:** Mesut Karatas, Cengiz Sabanoglu, Kader Eliz Sahin, Ibrahim Halil Inanc

**Affiliations:** 1 Department of Cardiology, Kosuyolu High Specialization Education and Research Hospital, Istanbul 34865, Turkey; mesut.cardio@gmail.com; 2 Department of Cardiology, Umraniye Education and Research Hospital, Istanbul 34760, Turkey; drchingiz23@gmail.com; 3 Department of Cardiology, Kocaeli City Hospital, Kocaeli 41060, Turkey; kesahin@yahoo.com; 4 Department of Internal Medicine, Phoenixville Hospital-Tower Health, Phoenixville, PA 19460, USA

**Keywords:** left ventricular global function index, performance, non-ST elevation myocardial infarction, major adverse cardiovascular events

## Abstract

*Background and Objectives*: The prognostic value of Left Ventricular Global Function Index (LVGFI) in chronic cardiovascular diseases is well-documented; however, limited evidence exists for its utility in non-ST elevation myocardial infarction (NSTEMI). This study aims to evaluate LVGFI as a predictor of three-year mortality and major adverse cardiovascular events (MACE) in NSTEMI patients. *Materials and Methods*: This retrospective cohort study included 432 NSTEMI patients divided into tertiles based on LVGFI values: T1 (low), T2 (intermediate), and T3 (high). LVGFI values were derived from echocardiographic imaging. Kaplan–Meier survival analysis was used to assess outcomes, and the Cox proportional hazards models, adjusted for demographics and clinical covariates, determined the association between LVGFI tertiles and three-year outcomes. *Results*: The average age and sex distribution were similar across tertiles with no significant differences in cardiovascular risk factors or most laboratory parameters. However, significant differences were noted in body surface area (higher in T3), platelet counts (higher in T1), and triglyceride levels (lower in T3). The ROC analysis identified an optimal LVGFI cut-off of 23.22 for predicting three-year mortality, with a sensitivity of 72% and specificity of 75% (AUC: 0.81; 95% CI: 0.74–0.87, *p* < 0.001). Patients in the T1 exhibited a three-year mortality rate of 25%, compared to 2.1% in the T3. After adjustment, the hazard ratio (HR) for mortality was significantly higher in T1 (HR 11.86; 95% CI: 3.60–39.10) compared to T3. Similarly, MACE rates were highest in T1 (27.1%) and lowest in T3 (7.6%). *Conclusions*: LVGFI is a significant independent predictor of three-year mortality and MACE in NSTEMI patients.

## 1. Introduction

Left ventricular dysfunction is a key determinant of prognosis in patients with non-ST elevation myocardial infarction (NSTEMI), a common and clinically significant form of acute coronary syndrome (ACS) [1]. Despite advances in treatment strategies, NSTEMI patients remain at high risk for long-term adverse cardiovascular events, including mortality and major adverse cardiovascular events (MACE) [2,3]. Effective risk stratification is essential for identifying high-risk individuals and tailoring management strategies to reduce morbidity and mortality. However, traditional risk markers, such as left ventricular ejection fraction (LVEF), while helpful, have limitations in the acute setting, as they may not fully capture the complexity of left ventricular (LV) performance, particularly in the early phases of myocardial injury [4]. Moreover, LVEF primarily reflects systolic function and may fail to detect subtle abnormalities in diastolic function or the progressive changes in ventricular remodeling that occur after NSTEMI [5]. These limitations have led to the investigation of novel indices for assessing myocardial function, such as LV global longitudinal strain (GLS), gaining recognition over the past decade as a promising alternative for evaluating LV systolic performance [6].

The Left Ventricular Global Function Index (LVGFI) is a novel composite marker that integrates multiple dimensions of LV function, including both volumetric and functional parameters. By combining stroke volume, LV total volume, and myocardial mass, LVGFI offers a more holistic assessment of cardiac performance, potentially providing more accurate prognostic information than traditional indices [7]. Previous studies have demonstrated the value of LVGFI in healthy populations, chronic cardiovascular diseases, including heart failure, hypertrophic cardiomyopathy, and chronic kidney disease, where it has shown superior predictive power for adverse outcomes compared to traditional measures like LVEF [7,8,9,10]. Despite these findings, the role of LVGFI in predicting outcomes in acute settings, particularly in NSTEMI, remains underexplored. Two recent studies highlighted the role of LVGFI in evaluating cardiac function and predicting outcomes in ACS patients, including those with NSTEMI [11,12]. Given the dynamic nature of myocardial stress and recovery in NSTEMI, a more comprehensive index like LVGFI may have unique advantages for identifying patients at higher risk for adverse events in the short and long term.

This study aims to evaluate the predictive value of LVGFI for three-year mortality and MACE in NSTEMI patients, hypothesizing that LVGFI offers superior prognostic information compared to traditional markers.

## 2. Materials and Methods

Ethical approval for this retrospective cohort study was obtained from the Ethics Committee of Mogadishu Somali Turkey Recep Tayyip Erdogan Education and Research Hospital, and records were obtained from the same hospital. The study was conducted in accordance with the principles outlined in the Declaration of Helsinki. The power analysis was conducted to assess the adequacy of the sample size for detecting differences in MACE rates. Based on the significance level of 0.05 and 80% power, the estimated required sample size was 174 patients per group (348 total). Our study included a total of 432 patients diagnosed with NSTEMI. Patients were categorized into three groups based on their LVGFI values: T1 (low), T2 (intermediate), and T3 (high), with each group consisting of 144 patients. Baseline demographic and clinical characteristics, including age, sex, body mass index (BMI), and comorbidities such as hypertension, diabetes mellitus, and smoking status, were recorded for all patients. Laboratory parameters, including glucose levels, lipid profiles, hemoglobin, platelet count, and creatinine levels, were also collected.

### 2.1. Recruitment of Patients

Patients eligible for inclusion in this study were those aged 18 years or older with a diagnosis of NSTEMI, confirmed by clinical presentation, elevated cardiac biomarkers (such as troponins or creatine kinase MB), and electrocardiographic findings indicative of ischemia without ST-segment elevation, who received standard care for NSTEMI, including medical management (e.g., antiplatelet therapy, anticoagulants, and statins) and invasive procedures (e.g., coronary angiography and percutaneous coronary intervention), provided they had complete follow-up data over a three-year period to assess clinical outcomes such as mortality and MACE. Of note, only those with complete clinical and echocardiographic data, including LVGFI measurements, were included. MACE was defined as cardiovascular death, myocardial infarction, target vessel revascularization (TVR), and stroke during follow-up.

Exclusion criteria included patients with ST-elevation myocardial infarction (STEMI) (n = 256), as it involves different pathophysiology and prognosis. Additionally, those with significant structural heart conditions including severe valvular diseases (n = 133)**,** hypertrophic cardiomyopathy (HCM) (n = 30), restrictive cardiomyopathy (n = 22), and dilated cardiomyopathy (DCM) (n = 212), or advanced heart failure (defined as NYHA Class III/IV symptoms with LVEF < 35% and persistant symptoms despite optimal medical therapy) (n = 101) were excluded, as these could confound the assessment of LV function. Patients with inadequate echocardiographic data due to poor image quality (n = 32), as well as those who could not provide informed consent due to cognitive impairment or other factors (n = 26), were also excluded. Other exclusions included patients with active inflammatory or infectious diseases, end-stage renal disease (ESRD) requiring dialysis, and missing or incomplete records, particularly those lacking LVGFI calculations or key follow-up data.

### 2.2. Collection of Clinical Data

Demographic information, anthropometric measurements, vital signs, and relevant laboratory and imaging findings were collected from the institutional electronic health records (EHR) and, when necessary, supplemented with paper-based medical charts. Body weight (Wt) and height were recorded at the time of the patient’s initial evaluation or hospital admission. Body mass index (BMI) was calculated using the standard formula: BMI = weight (kg)/height^2^ (m^2^). Body surface area (BSA) was estimated using the Mosteller formula: BSA (m^2^) = √[(height in cm × weight in kg)/3600]. Blood pressure (BP) was measured using an automated sphygmomanometer or a manual device, following standardized protocols. Systolic and diastolic BP values were recorded.

### 2.3. Transthoracic Echocardiography and Categorization of Tertiles

The LVGFI was estimated using a comprehensive echocardiographic protocol based on established guidelines, specifically the American Society of Echocardiography (ASE) recommendations for chamber quantification [13]. Transthoracic echocardiography was performed using advanced imaging techniques with a Philips EPIQ 7G ultrasound system and a high-frequency 1–5 MHz transducer. Measurements were obtained from parasternal long-axis, short-axis, and apical four-chamber views to ensure accuracy and reproducibility. To calculate LVGFI, left ventricular volumes were measured using the biplane method of disks (modified Simpson’s rule) from the apical four-chamber and two-chamber views. Left ventricular end-diastolic volume (LVEDV) and end-systolic volume (LVESV) were derived by tracing the endocardial border in both diastole and systole, with the papillary muscles excluded. Stroke volume (SV) was calculated as the difference between LVEDV and LVESV. To determine the left ventricular global volume, the mean of LVEDV and LVESV was added to the myocardial volume, which was derived by dividing the left ventricular mass (LVM) by the specific myocardial density (1.05 g/mL). The LVGFI was then computed using the formula:

$$LVGFI=\frac{SV}{LV\;Global\;Volume}\times 100$$

$$LV\;Global\;Volume=\frac{LVEDV+LVESV}{2}+Myocardial\;Volume$$

$$Myocardial\;Volume=\frac{LV\;Mass}{1.05}$$

Patients were categorized into tertiles based on LVGFI distribution within the study population, with T1 (low LVGFI) representing the lowest third, T2 (intermediate LVGFI) the middle third, and T3 (high LVGFI) the highest third.

### 2.4. Laboratory Data

Laboratory data were retrospectively collected from the institutional EHR and included hematological, biochemical, and inflammatory markers. The most recent laboratory values within the study period were extracted for each patient. Blood samples were obtained through venipuncture and analyzed in the hospital’s accredited clinical laboratory using standardized assays.

### 2.5. Statistical Analysis

Descriptive statistics were used to summarize baseline characteristics, with continuous variables presented as mean ± standard deviation and categorical variables as percentages. Between-group comparisons were made using one-way analysis of variance (ANOVA) for continuous variables and chi-square tests for categorical variables. The primary outcome measures were three-year mortality and MACE, with differences between groups assessed using Kaplan–Meier survival curves and log-rank tests.

To evaluate the independent associations between LVGFI tertiles and clinical outcomes, Cox proportional hazards models were used. Model 1 was unadjusted, while Models 2, 3, and 4 adjusted for various covariates, including demographic characteristics (age, sex, BMI), clinical factors (systolic blood pressure, heart rate, previous coronary artery disease), and laboratory values (creatinine, hemoglobin, CRP, LDL). Hazard ratios (HR) with 95% confidence intervals (CI) were reported for mortality and MACE. A *p*-value of <0.05 with a two-tailed *t*-test was considered statistically significant. Receiver Operating Characteristic (ROC) analysis was performed to evaluate the predictive ability of LVGFI for three-year mortality by plotting the sensitivity against 1-specificity at various cut-off values, with the area under the curve (AUC) used to quantify its discriminative performance.

## 3. Results

The total number of patient records examined was n = 1244, from which n = 812 were excluded based on the predefined criteria. After applying the exclusion criteria, a total of 432 patients with NSTEMI were included in the final analysis with a mean age of 68 years, and 65% were male. There were no significant differences in key cardiovascular risk factors, including the prevalence of hypertension, diabetes mellitus, and smoking status. BSA was higher in T3 compared to T1 and T2 (*p* = 0.003). Platelet counts were highest in comparison to T2 and T3 (*p* = 0.049). Triglyceride levels were lowest in T3 relative to T1 and T2 (*p* = 0.011). Other laboratory parameters, such as glucose, hemoglobin, and lipid profiles, were not significantly different between the groups (Table 1).

Table 2 highlights the hemodynamic and echocardiographic characteristics of patients across LVGFI tertiles. Systolic and diastolic blood pressures, as well as heart rate, were similar among the groups (*p* > 0.05). LVEF showed no significant differences between groups (*p* = 0.901). However, SV was significantly lower in T1 (*p* < 0.001). LVM and LVMI showed a decreasing trend from T1 to T3, with LVM highest in T1 and lowest in T3, and LVMI declining from T1 to T3 (both *p* < 0.001). Left ventricular end-diastolic diameter (LVEDD) and LVEDV were consistent across tertiles (*p* = 0.167 and *p* = 0.407, respectively), while LVESV was slightly higher in T1, nearing significance (*p* = 0.050).

**Figure 1:**
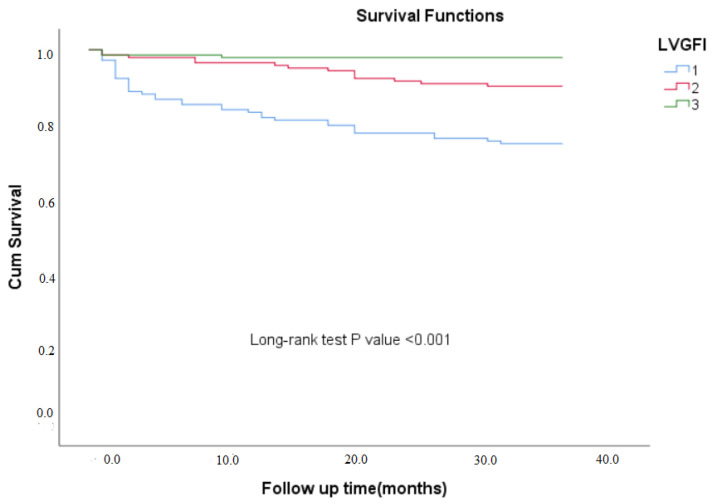
Kaplan–Meier survival analysis of three-year mortality across LVGFI tertiles.

Three-year clinical outcomes across the LVGFI tertiles (T1, T2, T3) were presented in Table 3. The mean follow-up duration was 35 months. The rates of TVR and recurrent myocardial infarction (RMI) were similar among the three tertiles, with TVR occurring in 3.5% of patients in both T1 and T2, and 4.2% in T3 (*p* = 0.937), while RMI rates were 6.3%, 3.5%, and 2.8%, respectively (*p* = 0.296). In contrast, significant differences were observed in the rates of MACE and mortality. MACE occurred in 27.1% of patients in T1, 14.6% in T2, and 7.6% in T3 (*p* < 0.001), while mortality was significantly more frequent in T1 (25.0%) compared to T2 (9.7%) and T3 (2.1%) (*p* < 0.001) (Figure 1).

Cox proportional hazards analysis for three-year mortality and MACE based on LVGFI tertiles was presented in Table 4. For three-year mortality, the unadjusted HR was highest in T1, with an HR of 14.45 (95% CI: 4.44–46.98) compared to T3. After adjusting for age, sex, BMI, systolic blood pressure, heart rate, and previous coronary artery disease (CAD), the HR remained significantly elevated in T1 (HR 11.86; 95% CI: 3.60–39.10), indicating a substantially higher risk of mortality. The risk of mortality in T2 was also significantly increased compared to T3, with HRs ranging from 4.86 (95% CI: 1.39–16.94) to 4.36 (95% CI: 1.23–15.47) across different models. For MACE, T1 also had the highest risk, with an unadjusted HR of 4.33 (95% CI: 2.21–8.48), and after adjustment for covariates, the HR remained elevated at 3.44 (95% CI: 1.71–6.90). The HR for MACE was significantly lower in T2, ranging from 2.02 (95% CI: 0.97–4.21) in the unadjusted model to 1.86 (95% CI: 0.87–4.01) after full adjustment, while T3 remained the reference group with the lowest event risk.

**Figure 2:**
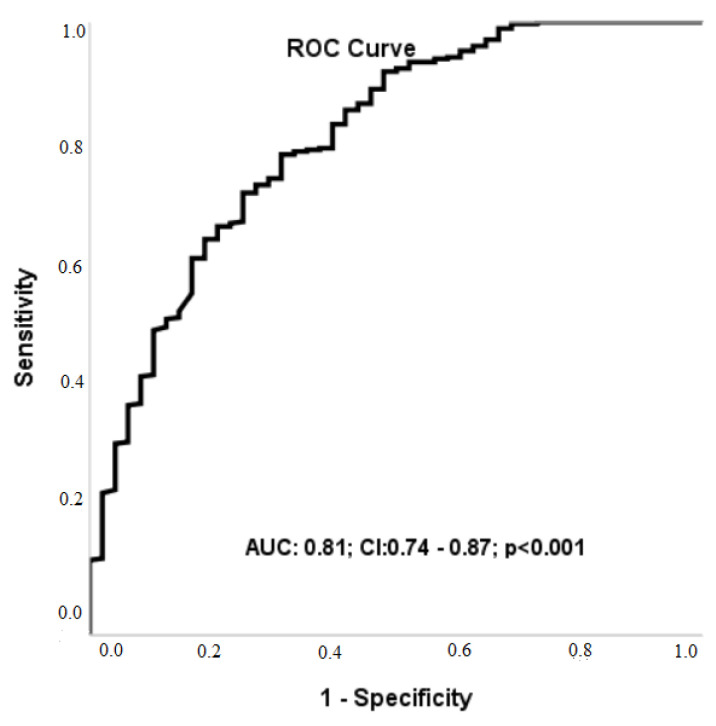
Receiver operating characteristic analysis of Left Ventricular Global Function Index for predicting three-year mortality.

The ROC analysis also demonstrated that an LVGFI cut-off value of 23.22 predicted three-year mortality with a sensitivity of 72% and specificity of 75% (AUC: 0.81; 95% CI: 0.74–0.87; *p* < 0.001) (Figure 2).

## 4. Discussion

In this study, our findings demonstrate that a lower LVGFI is significantly associated with higher mortality and MACE rates, underscoring its potential as a valuable prognostic tool in clinical practice.

In our study, the LVGFI was selected over traditional indices, such as LVEF, as it provides a more comprehensive assessment of cardiac function. Unlike LVEF, which primarily evaluates systolic function, LVGFI integrates multiple dimensions of left ventricular performance, encompassing volumetric and functional parameters [14]. The advantages of LVGFI also extend to its sensitivity in detecting early or subclinical left ventricular dysfunction, which is often missed by traditional metrics [7]. In a study, Diaz-Navarro et al. [5] characterized three patient groups, including acute myocarditis, takotsubo cardiomyopathy, and acute myocardial infarction, and found that the LVGFI offered incremental value over traditional metrics LVEF and GLS. Our study corroborates this advantage, as the LVEF did not differ significantly across tertiles, whereas LVGFI demonstrated significant predictive value for mortality and MACE.

Our results align with and extend the findings of several studies that have explored the prognostic value of LVGFI in ACS patients. Reinstadler et al. [15] demonstrated that LVGFI was a strong predictor of adverse events in patients with STEMI. In their study of 226 STEMI patients, they found that LVGFI independently predicted and had better prognostic value than traditional parameters such as LVEF. Eitel et al. [16] conducted a larger study with 795 STEMI patients and found that the LVGFI was strongly associated with markers of significant myocardial and microvascular injury in STEMI, providing superior prognostic value compared to traditional cardiac risk factors, including LVEF. Similarly to our study, Doganay et al. [11] evaluated the prognostic role of the LVGFI in predicting MACE in patients with acute coronary syndrome after 3-year follow-up. Decreased LVGFI levels were identified as independent predictors of MACE in both STEMI and NSTEMI groups.

LVGFI has demonstrated its utility in various clinical settings, extending beyond its application in acute coronary syndrome. Studies have explored its prognostic value in diverse patient populations, including those with chronic kidney disease (CKD), heart failure, hypertrophic cardiomyopathy (HCM), and amyloidosis [8,9,10,17,18]. For instance, Liu et al. [8] investigated the association between the LVGFI and clinical outcomes in patients with DCM. They found that lower LVGFI was linked to higher rates of death and heart failure events. Similarly, Schober et al. [18] revealed that in patients with implanted cardioverter-defibrillators (ICD) for secondary prevention, a reduced LVGFI was identified as an independent predictor of both mortality and rehospitalization. A prospective study including 158 patients with ESRD undergoing maintenance dialysis showed that a 10% decrease in LVGFI increased the risk of MACE by 114%, and the predictive model including LVGFI had significantly better performance in forecasting MACE compared to other cardiac parameters like native T1 mapping and GLS, with these findings remaining consistent even in patients with LVEF above the median [10]. Huang et al. [17] also demonstrated that LVGFI had excellent diagnostic performance in differentiating cardiac amyloidosis from HCM. Interestingly, a multicenter prospective cohort study evaluated the predictive value of the LVGFI for cardiovascular events in 5004 healthy participants with a median follow-up of 7.2 years. The results showed that LVGFI was significantly associated with heart failure, hard cardiovascular events, and all cardiovascular events, with lower LVGFI values independently predicting higher risk for these outcomes, highlighting its potential as a powerful prognostic tool in a multiethnic population without prior cardiovascular disease [7].

The primary distinction of our study lies in its focus on the prognostic value of LVGFI specifically within an acute NSTEMI patient population, whereas much of the existing literature has primarily explored LVGFI in more chronic or stable cardiovascular conditions. While LVGFI’s ability to capture subtle left ventricular dysfunction in these chronic settings is well-established, the pathophysiological dynamics in acute NSTEMI patients, characterized by rapid and significant myocardial stress, require distinct prognostic approaches. Our study contributes to the current literature by demonstrating LVGFI’s predictive value in an acute myocardial infarction context, thus broadening its clinical applicability. Notably, our findings highlight LVGFI’s strong association with three-year mortality and MACE, complementing and extending previous research and reinforcing its potential as a versatile prognostic tool across diverse cardiovascular conditions.

### Limitations

Despite these promising results, our study has limitations. First of all, the retrospective design and single-center setting may limit the generalizability of our findings. Second, we utilized echocardiographic measurements for LVGFI, which may have a lower degree of accuracy compared to MRI. However, this choice was deliberate, as LVGFI has the potential to be assessed using echocardiography, a more readily accessible imaging modality in the acute phase of NSTEMI. Third, a key limitation of our study is the reliance on volumetric calculations obtained through transthoracic echocardiography, which are subject to several constraints. These include dependency on the operator’s experience, the requirement for high-quality imaging to ensure optimal visualization of the endocardial border, and the inherent load-dependency of these measurements. Additionally, there is a potential for significant inter-reader variability, and external factors such as anterior chest wall deformities may further influence the accuracy of the assessments. Fourth, while we adjusted for several confounders, the possibility of residual confounding cannot be entirely excluded. Multi-center, prospective studies would be valuable in confirming and extending our results.

## 5. Conclusions

Our findings suggest that LVGFI is an independent predictor of three-year mortality and MACE in patients with NSTEMI, supporting its potential integration into clinical risk stratification models. Unlike traditional markers, LVGFI provides a holistic assessment of ventricular function, making it a valuable tool for identifying high-risk patients and guiding post-NSTEMI management. Future prospective studies are warranted to validate LVGFI’s clinical utility across diverse populations and to optimize its application in acute care settings.

## Figures and Tables

**Table 1:** Demographic, clinical, and laboratory characteristics of patients across LVGFI tertiles.

Characteristics	T1 (n = 144)	T2 (n = 144)	T3 (n = 144)	*p*-Value
Age (mean ± SD)	70 ± 12	67 ± 12	68 ± 13	0.238
Biological sex, male (n, %)	94 (65.3)	94 (65.3)	91 (63.2)	0.913
Body weight (mean ± SD)	75.2 ± 3.5	76.1 ± 3.6	80.5 ± 3.8	0.133
BMI (mean ± SD)	27.8 ± 3.9	27.5 ± 4.2	28.4 ± 3.9	0.121
BSA (mean ± SD)	1.78 ± 0.2	1.80 ± 0.2	1.86 ± 0.21	0.003
Hypertension (n, %)	83 (57.6)	85 (59.0)	80 (55.6)	0.835
Diabetes mellitus (n, %)	52 (36.1)	42 (29.2)	42 (29.2)	0.342
Smoking (n, %)	22 (15.3)	23 (16)	20 (13.9)	0.881
Previous CAD (n, %)	21 (14.6)	26 (18.1)	29 (20.1)	0.457
Sleep apnea (obstructive/central) (n, %)	29 (20)	36 (25)	43 (30)	0.220
**Laboratory parameters**				
Glucose, mg/dL (mean ± SD)	124 ± 44	117 ± 38	119 ± 46	0.407
Hemoglobin, g/dL (mean ± SD)	13.6 ± 2	13.8 ± 2	13.9 ± 2	0.483
White blood cell count, cells/µL (mean ± SD)	9.1 ± 3	9.2 ± 3	9.8 ± 3	0.085
Platelet count, cells/µL (median, percentile)	226 (190–269)	215 (181–256)	209 (172–263)	0.049
Lymphocyte count, cells/µL (mean ± SD)	2.3 ± 1	2.3 ±	2.5 ±	0.402
Neutrophil count, cells/µL (mean ± SD)	6.5 ± 2	5.9 ± 2	6.0 ± 2	0.063
Creatinine, mg/dL (mean ± SD)	0.98 ± 0.3	0.96 ± 0.3	0.97 ± 0.3	0.737
HDL (mean ± SD)	41.1 ± 11	46.2 ± 16	45.1 ± 15	0.166
LDL (mean ± SD)	118.4 ± 39	116.2 ± 35	116.4 ± 41	0.865
Triglyceride (median, percentile)	132 (99–176)	133 (85–186)	111 (81–154)	0.011
CRP (mean ± SD)	4.2 (1.9–11.0)	4.2 (1.9–10.2)	5.5 (2.4–13.0)	0.215

Abbreaviations: BMI: body mass index, BSA: body surface area, CAD: coronary artery disease, CRP: C-reactive protein.

**Table 2:** Hemodynamic and echocardiographic characteristics of patients across LVGFI tertiles.

Parameter (Mean ± SD)	T1 (n = 144)	T2 (n = 144)	T3 (n = 144)	*p*-Value
Systolic blood pressure, mmHg	131.7 ± 21.2	131.4 ± 21.4	129.4 ± 20.1	0.586
Diastolic blood pressure, mmHg	81.7 ± 15.7	81.4 ± 15.7	80.3 ± 16.4	0.747
Heart rate	74.3 ± 8.5	74.4 ± 8.3	73.3 ± 9.0	0.505
LVEF	54.53 ± 10.89	54.36 ± 11.04	53.95 ± 11.27	0.901
LVEDD	46.5 ± 4.62	47.44 ± 4.40	46.70 ± 4.34	0.167
LVEDV	86.66 ± 16.03	84.37 ± 13.76	85.87 ± 14.35	0.407
LVESV	45.51 ± 9.98	42.98 ± 10.42	45.65 ± 10.77	0.050
SV	57.43 ± 7.89	62.60 ± 7.67	65.17 ± 10.14	<0.001
LVM	216.22 ± 37.01	185.11 ± 39.79	176.13 ± 42.64	<0.001
LVMI	123.41 ± 26.92	103.59 ± 24.67	95.59 ± 24.64	<0.001

Abbreviations: LVEDD: left ventricular end-diastolic diameter, LVEDV: left ventricular end-diastolic volume, LVEF: left ventricular ejection fraction, LVESV: left ventricular end-systolic volume, LVM: left ventricular mass, LVMI: left ventricular mass index, SV: stroke volume.

**Table 3:** Three-year clinical outcomes of patients across the LVGFI tertiles.

	T1 (n = 144)	T2 (n = 144)	T3 (n = 144)	*p*-Value
TVR	5 (3.5)	5 (3.5)	6 (4.2)	0.937
RMI	9 (6.3)	5 (3.5)	4 (2.8)	0.296
Mortality	36 (25.0)	14 (9.7)	3 (2.1)	<0.001
MACE	39 (27.1)	21 (14.6)	11 (7.6)	<0.001

Abbreviations: MACE: major adverse cardiovascular events, RMI: recurrent myocardial infarction, TVR: target vessel revascularization.

**Table 4:** Cox proportional analysis for 3-year mortality and 3-year MACE by LVGFI tertiles.

	T1 (n = 144)	T2 (n = 144)	T3 (n = 144)
**3-year mortality**			
Number of deaths	36	14	3
Mortality, %	25	9.7	2.1
Mortality, HR (95% CI)			
Model 1: unadjusted	14.45 (4.44–46.98)	4.86 (1.39–16.94)	1[Reference]
Model 2: adjusted for age, sex, and BMI	14.18 (4.35–46.17)	4.85 (1.39–16.90)	1[Reference]
Model 3: adjusted for SBP, heart rate, and previous CAD	13.28 (4.08–43.26)	4.66 (1.33–16.27)	1[Reference]
Model 4: adjusted for all covariates ^a^	11.86 (3.60–39.10)	4.36 (1.23–15.47)	1[Reference]
**3-year MACE**			
Number of events	39	21	11
Events, %	27.1	14.6	7.6
Event, HR (95% CI)			
Model 1: unadjusted	4.33 (2.21–8.48)	2.02 (0.97–4.21)	1[Reference]
Model 2: adjusted for age, sex, and BMI	4.27 (2.18–8.35)	2.00 (0.96–4.15)	1[Reference]
Model 3: adjusted for SBP, heart rate, and previous CAD	4.04 (2.06–7.92)	1.94 (0.93–4.03)	1[Reference]
Model 4: adjusted for all covariates ^a^	3.44 (1.71–6.90)	1.86 (0.87–4.01)	1[Reference]

Abbreviations: SBP, systolic blood pressure; BMI, body mass index; CAD, coronary artery disease; HR, hazard ratio. ^a^ Includes demographics (age, gender and BMI); first measurement during hospitalization of the following laboratory values (creatinine, hemoglobin, CRP, LDL) and comorbidities (hypertension, diabetes mellitus, smoking, previous CAD, systolic blood pressure, heart rate).

## Data Availability

The datasets generated during and/or analyzed during the current study are available from the corresponding author upon reasonable request.
